# Supplementary material for: Backwashing performance of self-cleaning screen filters in drip irrigation systems
Source: PLoS One. 2019 Dec 19;14(12):e0226354. doi: 10.1371/journal.pone.0226354 (PMC6922369; doi:10.1371/journal.pone.0226354)
Supplement: Attachment — Submitted filename: Response to Reviewers.doc [file pone.0226354.s001.doc]

**Manuscript No.:** PONE-D-19-26474

**Title:** Backwashing Performance of Self-cleaning Screen Filters in Drip Irrigation Systems

**PLOS ONE**

The authors appreciate the efforts made by the reviewers and the Editor in providing the following comments and suggestions. We have addressed all of the comments. Revised portion are marked in red in the Revised Manuscript with Track Changes.

**Reply to 1’s Comments**

**Reviewer report:**

Reviewer #1: This is new contribution in the area of drip irrigation and adds new knowledge in filtering systems.

**Specific comments:**

**Question (1):** You may delete all introductory text leaving only findings and chapter objectives.

**Answer (1):**

Revised as recommended.

We deleted some introductory text in the instruction, including original Fig.1, text in the second and fifth paragraphs.

**Question (2):** Insert Figures and tables close to the text

**Answer (2):**

Revised as recommended. We inserted all figures and tables close to the text.

**Reply to Journal Requirements**

**Journal Requirements:** When submitting your revision, we need you to address these additional requirements.

Please ensure that your manuscript meets PLOS ONE's style requirements, including those for file naming. The PLOS ONE style templates can be found at http://www.journals.plos.org/plosone/s/file?id=wjVg/PLOSOne_formatting_sample_main_body.pdf andhttp://www.journals.plos.org/plosone/s/file?id=ba62/PLOSOne_formatting_sample_title_authors_affiliations.pdfReviewer's Responses to Questions

**Answer:**

Revised as recommended.

We revised the manuscript and ensured that it met PLOS ONE's style requirements, including the file naming, references etc.
